# Evidence for Benefits of Early Treatment Initiation for Chronic Hepatitis B

**DOI:** 10.3390/v15040997

**Published:** 2023-04-18

**Authors:** Young-Suk Lim, W. Ray Kim, Douglas Dieterich, Jia-Horng Kao, John F. Flaherty, Leland J. Yee, Lewis R. Roberts, Homie Razavi, Patrick T. F. Kennedy

**Affiliations:** 1 Department of Gastroenterology, Asan Medical Center, University of Ulsan College of Medicine, Seoul 05505, Republic of Korea; 2 Division of Gastroenterology and Hepatology, Stanford University School of Medicine, Palo Alto, CA 94063, USA; 3 Division of Liver Diseases, Icahn School of Medicine at Mount Sinai, New York, NY 10029, USA; 4 Division of Gastroenterology and Hepatology, Department of Internal Medicine, Department of Medical Research, Hepatitis Research Center, National Taiwan University Hospital, Taipei 100, Taiwan; 5 Graduate Institute of Clinical Medicine, National Taiwan University College of Medicine, Taipei 110, Taiwan; 6 Gilead Sciences, Foster City, CA 94404, USA; 7 Division of Gastroenterology and Hepatology, Mayo Clinic, Rochester, MN 55905, USA; 8 Center for Disease Analysis Foundation, Lafayette, CO 80026, USA; 9 Barts Liver Centre, Immunobiology, Blizard Institute, Barts and The London School of Medicine and Dentistry, Queen Mary University of London, London E1 4NS, UK

**Keywords:** hepatitis B, cirrhosis, hepatocellular carcinoma, liver fibrosis, viral hepatitis

## Abstract

Chronic hepatitis B (CHB) is the most common cause of hepatocellular carcinoma (HCC) worldwide. Antiviral treatment reduces the risk of HCC and mortality; nonetheless, globally in 2019, only 2.2% of CHB patients received treatment. Current international CHB guidelines recommend antiviral treatment only in subsets of patients with clear evidence of liver damage. This contrasts with hepatitis C or HIV where early treatment is recommended in all infected patients, regardless of end-organ damage. This narrative review aims to provide an overview of data on the early initiation of antiviral treatment and its related potential economic impact. Literature searches were performed using PubMed and abstracts from international liver congresses (2019–2021). Data on risk of disease progression and HCC and the impact of antiviral treatment in currently ineligible patients were summarized. Cost-effectiveness data on early antiviral treatment initiation were also collated. Accumulating molecular, clinical, and economic data suggest that early initiation of antiviral treatment could save many lives through HCC prevention in a highly cost-effective manner. In light of these data, we consider several alternative expanded treatment strategies that might further a simplified ‘treatment as prevention’ approach.

## 1. Introduction

In 2019, the World Health Organization (WHO) estimated that 296 million people had chronic hepatitis B virus (HBV) infection worldwide, resulting in an estimated 820,000 deaths annually, predominantly from cirrhosis and hepatocellular carcinoma (HCC) [1]. Chronic hepatitis B (CHB) is the most common cause of HCC, and rates of deaths from HBV-related HCC are expected to more than double between 2016 and 2040 [2].

A major goal of CHB antiviral treatment is to prevent disease progression, HCC, and mortality. Without a cure for CHB infection, an essential intermediate endpoint toward these goals is the long-term suppression of HBV replication, which is achievable by current antiviral treatment [3,4], and reduces the risk of HCC and mortality in CHB patients [3,5]. Nonetheless, globally, only 2.2% (6.6 million) of CHB patients received treatment in 2019 [1], due, in part, to the complex and restrictive clinical practice guidelines (Supplementary Figure S1). Currently, treatment is indicated only after the identification of hepatic necroinflammation through liver biopsy or in persons with specific elevations in the serum levels of alanine aminotransferase (ALT) and HBV DNA (Supplementary Figure S1) [3,4,6].

CHB is a dynamic disease, and viral load and risk of disease progression change over time due to the interaction between HBV replication and the host immune response [3,7]. Historically, chronic HBV infection has been divided into four disease phases (Supplementary File S1 and Supplementary Figure S1), with those that do not fall into these disease phases referred to as belonging to the ‘gray zone’. Different terminology is used to describe these phases, but for consistency, the terms immune tolerant (IT), immune active (IA), immune control (IC), and immune escape are used throughout this review. Terminology based on the European Association for the Study of the Liver (EASL) guidelines is included in the Supplementary File S1 and Supplementary Figure S1 for information [3]. Currently, the guidelines only recommend treatment in the IA and immune escape phases (Table 1).

This review examines the data supporting the earlier initiation of antiviral treatment to delay or even reverse CHB disease progression, and discusses the potential for ‘treatment as prevention’ as a strategy to reduce HBV-related mortality. We also collated the existing data on the potential economic impact of early antiviral treatment, as cost will be a key factor affecting implementation. Due to space restrictions, a comprehensive review of all the available data was not feasible. Further details of the search strategy and selection criteria are provided in the Supplementary File S2 and Supplementary Figure S2.

## 2. Mechanisms of Hepatocarcinogenesis in CHB Patients

**Figure 1:**
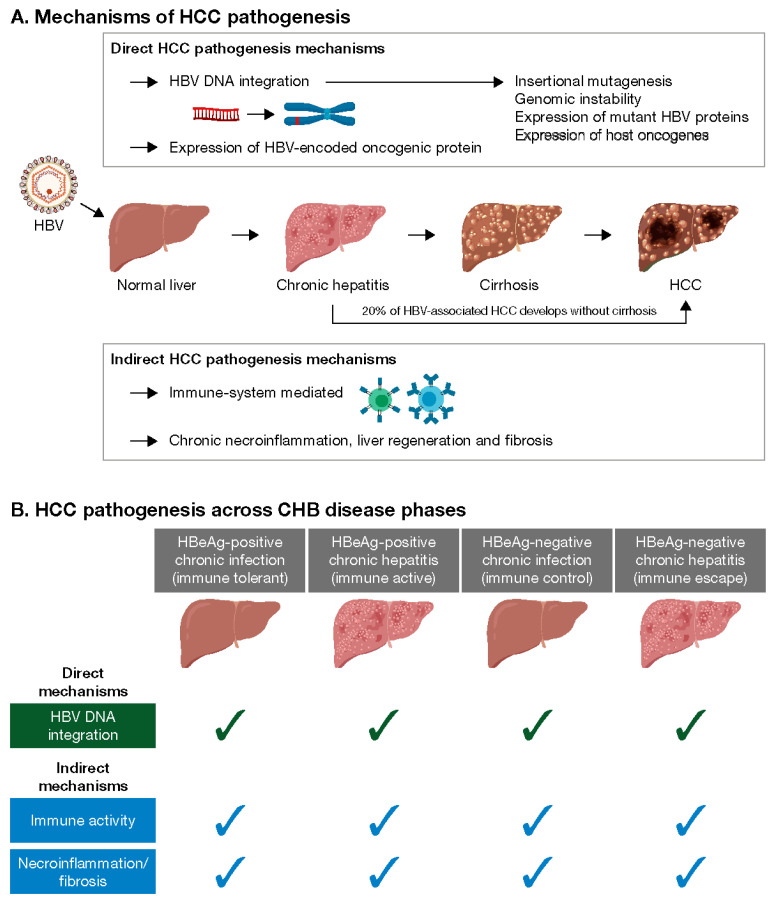
HCC pathogenesis. These schematics summarize some of the key HCC pathogenesis mechanisms in CHB patients (**A**) and in which CHB disease phases these have been detected (**B**). (**A**) HBV infects normal liver hepatocytes and can lead to CHB infection. During HBV infection, chronic hepatitis can develop, leading to cirrhosis and HCC in some patients. Approximately 20% of HBV-associated HCC cases develop in the absence of cirrhosis. HCC pathogenesis mechanisms can be direct or indirect. Direct HCC pathogenesis mechanisms are mediated by HBV and include HBV DNA integration into the host genome and the expression of HBV-encoded oncogenic protein. HBV DNA integration causes changes to the host genome via insertional mutagenesis, promoting genomic instability, and can lead to the expression of mutant HBV proteins. Indirect HCC mechanisms are mediated by the host immune system attacking HBV-infected hepatocytes. This leads to chronic necroinflammation, liver regeneration, and fibrosis, which cause genetic and epigenetic changes within hepatocytes. (**B**) Several studies have shown that HBV DNA integration into the host genome can be detected in liver samples from CHB patients across disease phases. Immune activity against HBV can also be detected in CHB patients across disease phases, as can necroinflammation and fibrosis in some patients. CHB, chronic hepatitis B; HBeAg, hepatitis B envelope antigen; HBV, hepatitis B virus; HCC, hepatocellular carcinoma.

During early HBV infection, the immune system is activated as part of the defense mechanism, and in acute cases, this response is beneficial. However, persistent immune activation from chronic infection initiates a series of molecular events [8] including carcinogenesis, driven via direct and indirect mechanisms (Figure 1) [5,9,10].

### 2.1. Direct Hepatocarcinogenesis

Direct hepatocarcinogenesis results from HBV DNA integration, which occurs prior to observable histological liver damage, and may promote HCC through chromosomal instability (including translocation), insertional mutagenesis, and the expression of mutant HBV genes or host oncogenes [5,9,11,12,13,14]. Integrations are detectable across multiple CHB phases (Figure 1) including the ‘gray zone’ [15,16,17,18], and correlate with levels of viremia [18]. Higher levels of integration have been observed among hepatitis B e antigen (HBeAg)-positive compared with HBeAg-negative patients [16], and among the HBeAg-negative patients, the highest number of integrations has been reported among those with HBV DNA >20,000 IU/mL [17]. Antiviral treatment has been shown to reduce the number of transcriptionally active integrations in patients with CHB [13,18,19].

Clonal expansion of hepatocytes containing integrations has been detected across all CHB phases [20,21], irrespective of HBeAg status or age [19]. It is possible that the immune response may select for hepatocytes with a survival advantage that clonally proliferate and initiate HCC, as although the integrations appear random, some occur in genes regulating cell proliferation and may drive hepatocarcinogenesis [17]. HCC can be polyclonal or monoclonal in origin; monoclonal tumors contain the same HBV DNA integration events, indicating that HBV DNA integration is an early driver in tumor development and remains stable during tumor progression [22].

### 2.2. Indirect Hepatocarcinogenesis

Indirect hepatocarcinogenesis is driven by chronic HBV-induced necroinflammation, regeneration, and fibrosis and can occur at any stage of disease [9,23,24]. HBV-specific T-cell activity is present in patients in both the IT and IA phases [21,24], and T-cell function has been shown to be similar, irrespective of the disease phase [25]. Analysis of baseline liver biopsies from treated CHB patients similarly found the immune microenvironment, classified as high (i.e., having elevated immune pathways and elevated immune cell signatures corresponding to B cells, T cells and macrophages) or low (not having these), to be independent of HBeAg status and HBV DNA levels [26].

## 3. Virological Risk Factors for HCC and Impact of Antiviral Therapy

### 3.1. Reappraisal of the Association between Viral Load and HCC Risk

Elevated HBV DNA is a strong risk factor for HCC and is a key consideration when deciding to initiate therapy in CHB patients [5]. Traditionally, HBeAg status has also been correlated with HCC risk [27,28]. However, as high viral loads can occur in HBeAg-negative patients and test sensitivity may affect its determination [29,30], HBeAg status should always be considered alongside the HBV DNA levels when estimating HCC risk.

**Figure 2:**
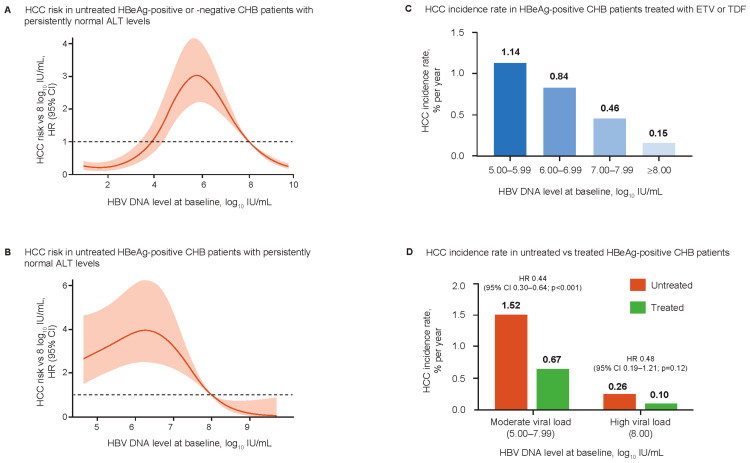
Association between the baseline HBV DNA levels and HCC risk in untreated and NA-treated non-cirrhotic adult patients with CHB. (**A**). In untreated HBeAg-positive and -negative, non-cirrhotic, adult CHB patients with persistently normal ALT levels (*n* = 6949), HCC risk was the highest with baseline levels of approximately 6 log_10_ IU/mL. (**B**). In untreated HBeAg-positive, non-cirrhotic, adult CHB patients with persistently normal ALT levels (*n* = 2081), HCC risk was the highest with baseline HBV DNA levels of approximately 6 log_10_ IU/mL. (**C**). In HBeAg-positive, non-cirrhotic, adult CHB patients treated with ETV or TDF (*n* = 2073), the on-treatment HCC incidence rate increased incrementally with decreasing baseline HBV DNA levels ≥5 log_10_ IU/mL. (**D**). Compared with untreated HBeAg-positive, non-cirrhotic adult CHB patients with normal ALT levels (*n* = 2643), NA treatment in HBeAg-positive, non-cirrhotic, adult CHB patients (*n* = 2073) reduced the HCC incidence in patients with moderate baseline viral load (5.00–7.99 log_10_ IU/mL), but the HCC risk did not decrease to the same extent in patients with a high baseline viral load (≥8.0 log_10_ IU/mL). ALT, alanine aminotransferase; CHB, chronic hepatitis B; CI, confidence interval; ETV, entecavir; HBeAg, hepatitis B envelope antigen; HBV, hepatitis B virus; HCC, hepatocellular carcinoma; HR, hazard ratio; NA, nucleos(t)ide analog; TDF, tenofovir disoproxil fumarate. All panels in this figure were made by Y-S Lim based on data in Kim G-A et al. *Aliment Pharmacol Ther.* 2020;51:1169–1179 (panels **A** and **B**) and Choi W-M et al. *J Clin Invest.* 2022;132:e154833 (panels **C** and **D**).

Although a linear association between baseline HBV DNA levels and HCC risk, regardless of ALT level or HBeAg status, has been observed with HBV DNA levels up to 6.0 log_10_ IU/mL [31,32,33], recent analyses suggest a more nuanced relationship [34,35,36]. Analysis of 6949 HBeAg-positive and -negative, non-cirrhotic, treatment-naïve CHB patients with ALT <80 U/L identified a parabolic association between HBV DNA levels and HCC risk (Figure 2A) [34]. Risk was highest among patients with HBV DNA levels of 6.0–7.0 log_10_ IU/mL and lowest in patients with HBV DNA levels ≤4 log_10_ IU/mL and >8.0 log_10_ IU/mL. This association was consistent across age groups, and neither HBeAg status nor ALT levels were predictive of HCC. A subsequent study analyzed the association between pre-treatment HBV DNA levels and HCC risk during treatment with entecavir (ETV) or tenofovir disoproxil fumarate (TDF) in 2073 HBeAg-positive, non-cirrhotic CHB patients [35]. At HBV DNA levels ≥5.0 log_10_ IU/mL, on-treatment HCC risk increased incrementally with decreasing baseline HBV DNA levels. By multivariable analysis, compared with baseline HBV DNA ≥8.0 log_10_ IU/mL, the adjusted hazard ratios for HCC risk for baseline HBV DNA 7.00–7.99 log_10_ IU/mL, 6.00–6.99 log_10_ IU/mL, and 5.00–5.99 log_10_ IU/mL were 2.48 (*p* = 0.03), 3.69 (*p* = 0.002), and 6.10 (*p* < 0.001), respectively (Figure 2B). Compared with untreated patients with the same ranges of baseline HBV DNA levels, antiviral treatment significantly reduced HCC risk in patients with moderate viral load (5.00–7.99 log_10_ IU/mL), but the HCC risk did not decrease to the level of patients who initiated antiviral treatment with a high viral load (≥8.0 log_10_ IU/mL; Figure 2C,D).

Moderate HBV DNA levels (10^5^–10^7^ IU/mL) are a risk factor for significant inflammation among patients with normal ALT and no significant fibrosis [36]. A fully infected liver can produce 10^9^–10^10^ virions/mL, which, if the infection is benign in an IT-phase host, would be expected to persist throughout the course of the disease [37]. Most HBeAg-positive CHB patients have very high HBV DNA levels (≥8.0 log_10_ IU/mL) during the initial phase of infection, and with a parabolic relationship, HCC risk may be relatively low [37]. Low and persistent immune-mediated damage to infected hepatocytes results in a gradual decrease in HBV DNA and progression to the moderate replication phase, which is associated with irreversibly increasing HCC risk. Accordingly, a decrease in viral load in untreated individuals may reflect progressive liver damage and increased HCC risk [37,38].

### 3.2. Impact of Antiviral Treatment

The current first-line nucleos(t)ide analogs (NAs), ETV, TDF, and tenofovir alafenamide (TAF), have favorable and well-described long-term safety profiles, with minimal to no resistance, even in heavily treatment-experienced patients with pre-existing resistance [3,4,6], and are associated with high adherence rates [39]. While long-term treatment does not eliminate HCC risk, it can reduce liver disease progression, improve necroinflammation and fibrosis, and even reverse cirrhosis [3,7,40,41,42].

However, CHB antiviral treatment indications and guidelines are largely based on evidence from randomized controlled trials (RCTs) that were not designed to demonstrate the impact of these treatments on long-term outcomes such as HCC in a real-world setting. As such, they may fail to address important groups of patients and result in the withholding of therapy in patients who may benefit. Moreover, the current concept of CHB disease phases and corresponding treatment guidelines based on HBeAg, HBV DNA, and ALT are complex and do not represent the whole spectrum of CHB patients observed in clinical practice. Consequently, many patients currently ineligible for NAs according to current guidelines remain at risk of adverse clinical events, particularly HCC. An analysis of treatment-naïve CHB patients showed that among those who developed HCC, 64%, 46%, and 34% did so outside the treatment guideline recommendations from the Asian Pacific Association for the Study of the Liver (APASL), American Association for the Study of Liver Diseases (AASLD), and EASL, respectively [43]. A similar analysis found that 75% of untreated patients who developed HCC were outside the AASLD guidelines [44].

## 4. Evidence for Risk of Disease Progression across CHB Disease Phases

### 4.1. Risks in Untreated, HBeAg-Positive CHB Patients in the IT Phase (Normal ALT and High HBV DNA Levels)

Contrary to conventional belief, IT patients may have significant liver injury or fibrosis [45,46]. Among 566 IT patients from 11 studies, 17% and 5% had significant fibrosis or advanced fibrosis, respectively [47]. Studies evaluating long-term outcomes in untreated patients in the IT phase have indicated a significant risk of HCC and adverse liver outcomes (Table 2). A multivariate analysis found that the risk of HCC and death or transplantation was significantly higher in untreated IT patients compared with NA-treated IA patients [48]. However, differences in 5- and 10-year cumulative HCC risk and liver cirrhosis progression were not observed between groups [49]. In another study, 3.7% of 651 IT patients developed HCC during a median 5.2 years of follow-up [50]. The 10-year HCC incidence rate was 2.6% and 20.4% in patients aged <40 years and ≥40 years, respectively. Finally, a retrospective multicenter study of 946 IT patients (mean HBV DNA 10^8.5^ IU/mL) reported a 10-year cumulative HCC risk of 1.7% [51]. Given that age and HBV DNA levels are important determinants of HCC risk, caution is required when interpreting HCC risk in these studies because the patients had a heterogeneous distribution of age and HBV DNA levels. Nonetheless, collectively, these data support the emerging consensus that the IT phase is not always benign, particularly among patients aged ≥40 years [48,49,50,51].

### 4.2. Risks in Untreated, HBeAg-Negative CHB Patients in the IC Phase (Normal ALT and Low HBV DNA Levels)

While CHB patients in the HBeAg-negative IC phase are generally ineligible for antiviral treatment, evidence suggests they have a higher risk of HCC or liver-related death compared with hepatitis B surface antigen (HBsAg)-negative controls [52]. Moreover, HBeAg-negative CHB patients are a heterogeneous population with respect to the risk of HCC and adverse liver outcomes (Table 2). An individual patient’s HCC risk depends on a combination of direct and indirect hepatocarcinogenesis mechanisms based on their unique disease history. For example, in a prospective observational study of 1192 patients with untreated HBeAg-negative CHB and low viral loads (mean baseline HBV DNA <10^4^ IU/mL), although the HBV DNA level correlated with disease progression, overall disease progression was minimal after 7 years, with no changes in fibrosis or HCC incidence [53]. In contrast, several other studies have reported substantial disease progression in IC patients. One study found an HCC incidence of 5% over a mean follow-up of 63 months among 337 treatment-naïve IC patients [54]. Another analysis of 7977 untreated IC patients found that annual cirrhosis and HCC incidence ranged from 0.3 to 1.3% and 0.04 to 3.8%, respectively [55]. A further study found that HCC occurred in 1.1% of 1014 untreated IC patients over a median follow-up of 42 months [56]. HCC occurred in 1.1% of inactive patients and in 7.7% of the treated patients.

**Table 2:** HCC risk in CHB patients in HBeAg-positive IT, HBeAg-negative IC, and ‘gray zone’ disease phases.

Author and Year	Study Type	Patient Population	HCC Risk
**HBeAg-positive IT disease phase**			
Kim 2018 [48]	Historical cohort study	Untreated IT (*n* = 413): HBV DNA ≥20,000 IU/mL and ALT < 1× ULN * (mean age 38 years, median HBV DNA 10^8^ IU/mL) vs. NA-treated IA (*n* = 1497): HBV DNA ≥20,000 IU/mL and ALT > 2× ULN * (mean age 40 years, median HBV DNA 10^8^ IU/mL)	10-year estimated cumulative HCC incidence: Untreated IT: 12.7% NA-treated IA: 6.1%; *p* = 0.001 Multivariate analyses showed: Untreated IT group had higher HCC risk vs. NA-treated IA group ○ HR = 2.54 (95% CI 1.54–4.18); *p* < 0.001 Older age was independently associated with higher HCC risk ○ HR = 1.08 (95% CI 1.06–1.11); *p* < 0.001
Kwon 2019 [49]	Multicenter cohort study	Untreated IT (*n* = 522): HBV DNA > 10^6^ IU/mL and ALT < 80 U/L (mean age 36 years) vs. NA-treated IA (*n* = 609): HBV DNA > 10^6^ IU/mL and ALT > 80 U/L (mean age 41 years)	5- and 10-year cumulative HCC risk Untreated IT: 0.3% and 1.3% NA-treated IA: 0.9% and 3.0%; *p* = 0.460 Age > 30 years was a significant risk factor in untreated IT group
Seong 2022 [50]	Retrospective cohort study	Untreated IT (*n* = 651): HBV DNA > 10^7^ IU/mL and ALT < 80 U/L (median age 36 years, median HBV DNA 10^8^ IU/mL)	After a median follow-up of 5.2 years, 3.7% of patients developed HCC Patients who developed HCC were significantly older than those who did not (49 years vs. 35 years; *p* < 0.001) 5-year HCC incidence rate: <40 years: 0% ≥40 years: 3.1% 10-year HCC incidence rate: <40 years: 2.6% ≥40 years: 20.4%
Lee 2020 [51]	Multicenter, retrospective cohort study	Untreated IT (*n* = 946): HBV DNA > 20,000 IU/mL and ALT ≤ 40 U/L (mean age 37 years, mean HBV DNA 10^9^ IU/mL)	10-year cumulative HCC risk Untreated IT: 1.7% Patients who developed HCC were significantly older than those that did not (51 years vs. 37 years; *p* = 0.001)
**HBeAg-negative IC disease phase**			
Chen 2010 [52]	Retrospective analysis of REVEAL-HBV cohort	Untreated IC (*n* = 1932): HBV DNA < 1900 IU/mL and ALT < 45 U/L (mean age 47 years) vs. uninfected controls (*n* = 18,137; mean age 46 years)	Annual HCC incidence rate: Untreated IC: 0.06% Uninfected controls: 0.02% Multivariate analysis showed untreated IC group had higher risk of HCC vs. uninfected controls: HR = 4.6 (95% CI 2.5–8.3) Older age was a significant HCC risk factor in both groups
Cho 2014 [56]	Retrospective study	Untreated IC (*n* = 1014): HBV DNA < 2000 IU/mL and ALT ≤ 40 IU/mL (mean age 52 years, mean HBV DNA 10^2^ IU/mL) vs. NA-treated (*n* = 1378): HBeAg positive, HBV DNA ≥20,000 IU/mL, ALT ≥ 2× ULN ^†^ or HBeAg negative, HBV DNA ≥2000 IU/mL, ALT ≥ 2× ULN ^†^ or compensated cirrhosis, HBV DNA ≥2000 IU/mL, any ALT or decompensated cirrhosis, any ALT (mean age 48 years, mean HBV DNA 10^6^ IU/mL) NA-treated patients with HBV DNA < 2000 IU/mL during follow-up were classified as complete responders (CR; *n* = 1132)	HCC incidence after median follow-up of 42 months: Untreated IC: 1.1% NA-treated: 7.7% NA-treated CR: 6.2% Annual HCC incidence rate: Untreated IC: 0.3% NA-treated CR: 2.3% 5-year cumulative HCC incidence: Untreated IC: 1.5% NA-treated CR: 11.4%
Seo 2020 [54]	Single-center study	Untreated IC (*n* = 337): HBV DNA < 2000 IU/mL and ALT ≤ 40 U/L (mean age 49 years, mean HBV DNA 309 IU/mL)	After a mean follow-up of 63 months, 4.5% of patients developed HCC Patients who developed HCC were significantly older than those who did not (62 years vs. 56 years; *p* = 0.035)
Liu 2021 [55]	Retrospective analysis of REAL-B registry	Untreated IC ^‡^ (*n* = 7977) vs. untreated IA ^‡^ (*n* = 549)	Annual HCC incidence: Untreated IC: 0.04–3.80% Untreated IA: 0.19–6.03%
**‘Gray zone’ or indeterminate disease phase**			
Huang 2022 [57]	Retrospective cohort study	Non-cirrhotic, untreated patients (*n* = 3366) classified by disease phase ^¶^ at baseline (inactive [*n* = 1370], indeterminate [*n* = 1303], IA [*n* = 481], IT [*n* = 212])	By up to 10 years of follow-up, of the 1303 indeterminate patients: 686 (52.7%) remained indeterminate 283 (21.7%) transitioned to the IA phase 314 (24.1%) transitioned to the inactive phase 20 (1.5%) transitioned to the IT phase Persistently indeterminate vs. persistently inactive patients: 10-year cumulative HCC incidence: 4.6% (95% CI 3.0–7.2) vs. 0.5% (95% CI 0.2–1.3); *p* < 0.0001 Risk of HCC development (multivariate analysis): adjusted HR = 14.1 (95% CI 1.3–153.3); *p* = 0.03
Tseng 2021 [58]	Retrospective analysis of ERADICATE-B cohort	Patients (*n* = 2150) stratified by HBV DNA levels (<2000 IU/mL/2000–<20,000 IU/mL/≥20,000 IU/mL) and ALT levels (≤ULN ^§^/1–2× ULN/≥2× ULN)	HCC risk per ‘treatment grey zone’ group compared with HBV DNA <2000 IU/mL and ALT ≤ ULN group: HBV DNA < 2000 IU/mL and ALT 1–2× ULN: HR = 4.07 (95% CI 1.92–8.64); *p* < 0.001 HBV DNA <2000 IU/mL and ALT ≥ 2× ULN: HR = 5.12 (95% CI 1.97–13.32); *p* = 0.001 HBV DNA 2000–<20,000 IU/mL and ALT ≤ ULN: HR = 2.27 (95% CI 1.16–4.46); *p* = 0.017 HBV DNA 2000–<20,000 IU/mL and ALT 1–2× ULN: HR = 6.69 (95% CI 2.95–15.20); *p* < 0.001 HBV DNA ≥20,000 IU/mL and ALT ≤ULN: HR = 5.18 (95% CI 2.80–9.59); *p* < 0.001
Choi 2019 [59]	Historical cohort study	Untreated inactive phase (*n* = 3572): HBV DNA < 2000 IU/mL and ALT < ULN ^†^ (mean age 47 years, median HBV DNA undetectable) Untreated replicative phase (*n* = 900): HBV DNA ≥2000 IU/mL and ALT < ULN ^†^ (mean age 47 years, median HBV DNA 10^4^ IU/mL) Untreated mildly active phase (*n* = 396): HBV DNA ≥2000 IU/mL and ALT 1–<2× ULN ^†^ (mean age 46 years, median HBV DNA 10^5^ IU/mL) NA-treated active phase (*n* = 546): HBV DNA ≥2000 IU/mL and ALT ≥ 2× ULN ^†^ (mean age 47 years, median HBV DNA 10^7^ IU/mL)	HCC cases per 100 patient-years (95% CI): Untreated inactive phase: 0.41 (0.35–0.48) Untreated replicative phase: 0.96 (0.76–1.23) Untreated mildly active phase: 1.94 (1.52–2.48) Treated active phase: 1.32 (1.02–1.71) Multivariate analysis showed that untreated replicative and mildly active phase groups had higher risk of HCC vs. NA-treated active phase group: Untreated replicative phase: HR = 1.47 (95% CI 1.01–2.15); *p* = 0.045 Untreated mildly active phase: HR = 2.02 (95% CI 1.41–2.91); *p* < 0.001

* ULN defined as <30 U/L for males and <19 U/L for females; ^†^ ULN defined as 40 U/L; ^‡^ Defined according to APASL guidelines; ^§^ ULN defined as 35 U/L for males and 25 U/L for females; ^¶^ Defined according to the AASLD guidelines. AASLD, American Association for the Study of Liver Diseases; ALT, alanine aminotransferase; APASL, Asian Pacific Association for the Study of the Liver; CI, confidence interval; CHB, chronic hepatitis B; CR, complete responders; HBeAg, hepatitis B envelope antigen; HBV, hepatitis B virus; HCC, hepatocellular carcinoma; HR, hazard ratio; IA, immune active; IC, immune control; IT, immune tolerant; NA, nucleos(t)ide analog; ULN, upper limit of normal.

### 4.3. Risks in Untreated ‘Gray Zone’ Patients (HBV DNA ≥2000 IU/mL and Minimally Raised Serum ALT Levels)

Current treatment criteria leave many patients in an untreated ‘gray zone’ or ‘indeterminate’ phase, despite evidence showing them to have increased HCC risk (Table 2). One study of 3366 untreated, non-cirrhotic CHB patients found that of the 38.7% classified as being in an ‘indeterminate’ phase at the baseline, 52.7% remained indeterminate 10 years later [57]. Compared with patients who remained in an inactive phase across the study period, these patients were found to have a 14 times higher risk of developing HCC. Similarly, an analysis of 2150 untreated, HBeAg-negative, non-cirrhotic CHB patients classified as being in the ‘gray zone’ found that these patients had an increased HCC risk [58]. Another study reported HCC in untreated ‘inactive phase’ patients (0.41 cases per 100 patient-years) [59]. This rate is similar to the incidence reported in the REVEAL-HBV study in patients with HBV DNA levels of 2000–20,000 IU/mL (0.30 per 100 patient-years) [31]. Again, caution is needed when comparing these studies as higher baseline HBV DNA levels (up to 10^6^ IU/mL) are associated with increasing HCC risk in HBeAg-negative CHB patients, and patients included in these studies had a heterogeneous distribution of baseline HBV DNA levels.

Collectively, these data provide evidence that patients ineligible for treatment (Table 1) remain at risk for HCC, and that this risk increases with age and increasing HBV DNA levels, regardless of ALT. Serum ALT is commonly used as a surrogate marker of liver injury [60] and plays a role in defining CHB disease phases (Supplementary Figure S1) and indications for treatment (Table 1) [3,4,6,61]. However, natural fluctuations in ALT and limited sensitivity and specificity in reflecting hepatic necroinflammation compromise its use in predicting disease progression, and evidence suggests CHB patients with persistently normal or minimally elevated ALT remain at risk of liver damage (Supplementary Table S1). Given that modern antiviral agents can competently control the low-level viremia seen in ‘gray zone’ patients, initiation of NA treatment may minimize the risk of disease progression and HCC in these patients, who are currently outside treatment indications.

## 5. Potential Impact of Early HBV Treatment on Hepatocarcinogenesis and Clinical HBV Parameters

### 5.1. Potential Impact of HBV Treatment on HBV DNA Integration

The intermediary form of HBV prior to integration into the host DNA contains double-stranded linear DNA (dslDNA). As dslDNA is formed via the reverse transcription of HBV RNA [62], NAs should reduce the formation of dslDNA and DNA integration into the host genome. While data on antiviral treatment and HBV DNA integration are limited, some evidence suggests that NA therapy may reduce hepatocarcinogenesis. Analysis of treated and untreated liver biopsies has demonstrated that treatment is associated with reductions in viral load, integrations [13,18], and chromosomal translocations [13]. Further investigation of the effect of antiviral treatment on HBV DNA integrations is required; however, these emerging data provide support for early CHB treatment with respect to HBV DNA integration and reducing HCC risk.

### 5.2. Potential Impact of Early HBV Treatment on Clinical HBV Parameters

**Figure 3:**
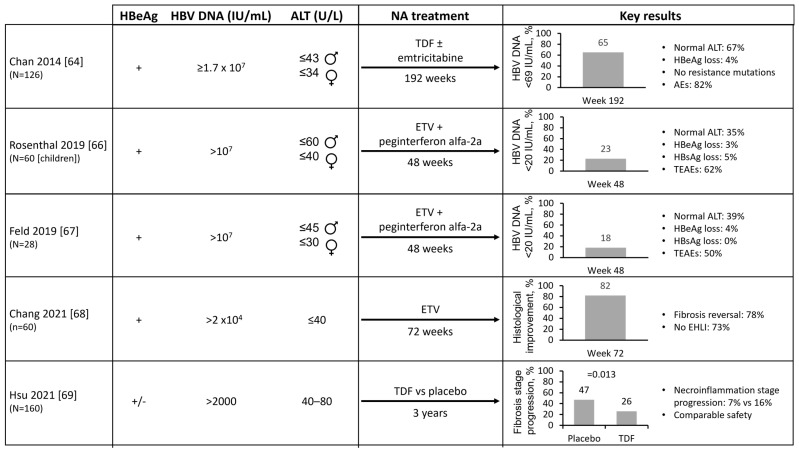
Impact of NA treatment on CHB clinical parameters in patients currently ineligible for antiviral treatment. This schematic summarizes the studies of NA treatment in CHB patients currently ineligible for antiviral treatment including those in the HBeAg-positive chronic infection/immune-tolerant disease phase. Patient numbers are shown in brackets in the first column. Patient baseline characteristics (HBeAg status and HBV DNA and ALT level) are shown in the second column. NA treatment and duration are shown in the third column. Key efficacy and safety results are shown in the fourth column. AE, adverse event; ALT, alanine aminotransferase; CHB, chronic hepatitis B; EHLI, evident histological liver injury; ETV, entecavir; HBeAg, hepatitis B envelope antigen; HBsAg, hepatitis B surface antigen; HBV, hepatitis B virus; NA, nucleos(t)ide analog; TDF, tenofovir disoproxil; TEAE, treatment-emergent adverse event [64,66,67,68,69].

A small number of studies have evaluated the impact of antiviral treatment on the virological, serological, and liver-related outcomes in CHB patients ineligible for treatment under the current guidelines (Figure 3). Data on long-term outcomes including HCC are not available, and RCTs comparing antiviral treatment to no treatment have not been performed. However, a meta-analysis that included two studies of IT patients found moderate-quality evidence for improved intermediate outcomes (viral suppression, HBeAg seroconversion/loss) with antiviral therapy [63]. Additionally, a Phase 2 study of TDF ± emtricitabine treatment in 126 IT patients found that 65% of patients had HBV DNA <69 IU/mL after 192 weeks of treatment, with 42% of patients with a moderate aMAP (age, male, albumin-bilirubin, and platelets) risk score at baseline shifting to the low-risk category with no HCC reported [64,65]. However, only 4% and 0% of patients had HBeAg or HBsAg loss, respectively. Based on these results, the authors concluded that routine NA treatment of patients with IT CHB is not warranted, as reflected in the current guidelines.

A multicenter study investigating ETV + peginterferon alfa-2a treatment in 60 children with IT CHB found that 75% had HBV DNA ≤1000 IU/mL and 23% had HBV DNA <20 IU/mL after 48 weeks of treatment, with HBeAg and HBsAg loss in two patients [66]. Another study of ETV + peginterferon alfa-2a treatment in 28 adult IT patients reported HBV DNA ≤1000 IU/mL in 93% of patients and HBV DNA <20 IU/mL in 18% of patients after 48 weeks [67]. In both studies, HBV DNA levels increased following discontinuation [66,67]. Analysis of 181 treatment-naïve IT CHB patients, where 33% of patients had evident histological liver injury (EHLI) at baseline, reported histological improvement and fibrosis reversal in 82% and 78% of patients with EHLI, respectively, following 72 weeks of ETV treatment, with 73% of patients no longer having EHLI [68].

### 5.3. Impact of Treatment on ‘Gray Zone’ Patients

Evidence for the treatment of ‘gray zone’ patients was provided by an analysis from the TORCH-B study, a randomized, double-blind, placebo-controlled study examining the treatment of patients with HBV DNA >2000 IU/mL, ALT 40–80 U/L, and no cirrhosis (79% HBeAg negative) [69]. During 3 years of follow-up, the placebo group showed a significantly higher proportion of progression in the fibrosis stage compared with the TDF group (47% vs. 26%; *p* = 0.013).

### 5.4. Impact of Early HBV Treatment in Patients with HBV/HIV Co-Infection

A unique group of CHB patients who routinely receive early antiviral treatment are those co-infected with human immunodeficiency virus (HIV). Indeed, provision of antivirals as pre-exposure prophylaxis to individuals at high risk of infection is considered as an important step in HIV control [70]. Many antiretroviral regimens include an NA component (TDF or TAF) and are initiated irrespective of the HBV DNA or ALT levels [3,4]. Consequently, the analysis of HCC risk in these patients can provide data about the potential impact of early antiviral treatment, with the major caveat that these are not RCTs. Among the 3625 HBV/HIV co-infected patients, the HCC incidence remained stable in those on NA treatment, but increased among patients receiving a regimen not including an NA [71]. Results from a study of antiviral-treated HBV mono-infected (*n* = 53,974) and HBV/HIV co-infected (*n* = 822) patients demonstrated lower HCC among HBV/HIV co-infected patients vs. HBV mono-infected patients [72]. Similarly, the analysis of claims data found lower HCC rates in HBV/HIV co-infected patients (*n* = 7764) compared with HBV mono-infected patients (*n* = 13,964) [73]. Assuming HIV co-infection is a proxy for early HBV antiviral treatment, these data suggest that the universal antiviral treatment of CHB patients may reduce the HCC risk.

## 6. Cost Effectiveness of Expanded HBV Treatment Strategies

Several studies have estimated that the costs associated with expanded CHB therapy may be offset by reduced expenditure needed for future consequences of disease progression from untreated CHB (Table 3).

A recent Chinese study that modeled the cost effectiveness of 136 expanded treatment strategies found treating all HBsAg-positive patients aged 18–80 years with a treatment coverage of 80% to be the most cost-effective strategy [74]. This strategy was predicted to prevent 82.0% of HBV-related complications by 2050, although a treatment coverage of ≥60% was considered sufficient to achieve the WHO goal of a 65% reduction in CHB-related mortality by 2030. Importantly, lowering the treatment initiation threshold was found to be more effective in preventing CHB-related complications than increasing the treatment coverage. In light of this, the Chinese hepatology and infectious diseases societies have revised their guidelines for the prevention and treatment of CHB, recommending antiviral treatment in all HBsAg-positive patients with detectable serum HBV DNA (i.e., HBV DNA 10–20 IU/mL) over the age of 30 years, regardless of ALT level [75].

Other studies from France and the U.S. have shown that expanding treatment to all CHB patients, regardless of fibrosis level and disease phase, to be the most cost-effective strategy in terms of clinical outcomes [76,77]. Furthermore, an economic impact analysis found that this strategy had a lower overall cost than continuing with the current guidelines, as fewer patients were lost to follow-up or presented with advanced liver disease [78]. Similarly, a Korean study reported that starting treatment in the IT phase was more cost effective than delaying until the IA phase [79]. However, a UK study found that while treating all HBeAg-negative patients was the most cost-effective strategy, for HBeAg-positive patients, it was more cost effective to only treat those with fibrosis stage 2 and above [80].

Studies from Korea have compared the current guidelines with an extended indication (all patients with HBV DNA ≥2000 IU/mL and any ALT). Under this strategy, HCC risk decreased by 1%, 2%, and 6% per 10% increase in the treatment uptake rate under the Korean National Health Insurance, EASL, and extended indications, respectively [81]. This approach was estimated to be highly cost effective and would be most impactful when 70% of patients with HBV DNA ≥2000 IU/mL were treated, regardless of ALT and HBeAg status [82].

However, a study from Saudi Arabia found that the treatment costs needed to be reduced in order for expanded treatment strategies to achieve a positive return on investment (ROI) [83]. Compared with the scenario of no policy change, they reported that the WHO target strategy (diagnose 90% of infections and treat 80% of high viral load patients by 2030) would lead to a 30–35% reduction in HCC and liver-related deaths, while a diagnose-and-treat-all strategy (diagnose and treat all infected patients by 2022) would lead to a 50–55% reduction by 2030. Achieving the WHO targets was estimated to achieve a ROI by 2021; however, the diagnose-and-treat-all strategy would require at least 50% lower treatment costs to achieve a ROI by 2028.

**Table 3:** Cost effectiveness and clinical impact of the expanded HBV treatment strategies.

Author and Year	Model Population and Selected Input Parameters	Treatment Strategies	Key Results
Zhang 2023 [74]	Chinese model of 136 scenarios based on cross combination of: ALT treatment initiation thresholds (40 U/L, 35 U/L for males and 25 U/L for females, 30 U/L for males and 19 U/L for females, and treating HBsAg-positive individuals regardless of ALT values) Population age groups (18–80, 30–80, and 40–80 years) Implementation durations (2023, 2028, and 2033) Treatment coverages (20%, 40%, 60%, and 80%)	Base case: status quo in China 135 treatment-expanding scenarios	Treating all HBsAg-positive individuals with 80% coverage was the most effective strategy for reducing HBV-related complications This strategy was also the most cost effective; however, all expanded treatment strategies were cost effective by 2050
Lepers 2020 [76]	CHB patients in French ANRS CO22 HEPATHER cohort HBeAg-positive chronic infection: 1% ○ F0–F3: 30 years, HCC transition probability = 0.001 ○ F4: 54 years, HCC transition probability = 0.001 HBeAg-positive chronic hepatitis: 6% ○ F0–F3: 36 years, HCC transition probability = 0.001 ○ F4: 54 years, HCC transition probability rate = 0.019 HBeAg-negative chronic infection: 56% ○ F0–F3: 42 years, HCC transition probability = 0.001 ○ F4: 54 years, HCC transition probability = 0.017 HBeAg-negative chronic hepatitis: 36% ○ F0–F3: 42 years, HCC transition probability = 0.001 ○ F4: 54 years, HCC transition probability = 0.030	Follow current treatment recommendations Treat patients in chronic hepatitis disease phase regardless of fibrosis stage Treat patients with ≥F2 fibrosis score regardless of disease phase Treat all patients	Treat all patients was the most expensive and cost-effective strategy and the most effective in terms of clinical outcomes
Razavi-Shearer 2021 [77]	U.S. model including historical and future impact of immigration using 164 country-specific disease burden and transmission models	Base case: current treatment strategy Treat all: treat all HBsAg-positive individuals	Disease burden outputs through 2050 compared with base case: ○ Treat-all strategy: ↓ 10,000 CHB cases, ↓ 49,000 decompensated cirrhosis cases, ↓ 132,000 HCC cases, ↓ 157,000 deaths Cost effectiveness of treat-all strategy compared with base case: ○ Current estimated annual NA price (USD$5400) → cost effective ○ Annual NA price USD$2000 → highly cost effective ○ Annual NA price USD$750 → cost saving
Razavi 2020 [78]	Economic impact analysis	Test and treat all HBsAg-positive individuals Treat according to current guidelines	Treating according to the currently guidelines was more expensive than test-and-treat-all strategy due to loss to follow-up and presentation with advance liver disease
Kim 2021 [79]	Hypothetical CHB patients 35 years of age HBeAg positive Mean HBV DNA 10^7.6^ IU/mL Normal ALT Non-cirrhotic Annual transition probabilities to HCC: Treat IT: 0.0033 Untreated IT: 0.0073	Treat IT: start treatment at IT phase Untreated IT: delay treatment until immune-active phase	Treat IT strategy was increasingly cost effective compared with untreated IT strategy Inclusion of lost productivity costs showed that treat IT strategy was dominant, with ICER below 0 in most cases
Crossan 2016 [80]	Hypothetical CHB patients with suspected fibrosis (*n* = 1000) Separate Markov models constructed for HBeAg-positive (starting age 31 years) and -negative (starting age 40 years) patients Transition probabilities: Moderate fibrosis → HCC: 0.048 Cirrhosis → HCC: 0.024	Treat all without fibrosis assessment Biopsy all patients and treat those with fibrosis stage ≥2 Test all patients with non-invasive test and treat those with fibrosis stage ≥2 Treat no patients	HBeAg-negative patients: treat all patients was the most cost-effective strategy HBeAg-positive patients: test all patients using FibroScan and treat those with fibrosis stage ≥2 was the most cost-effective strategy
Shim 2016 [81]	CHB patients in Korea Mean age: 56 years HBeAg positive: 66% HBV DNA > 10^4^ IU/mL: 48% Mean ALT: 30 U/L Non-cirrhotic HCC risk predicted using REACH-B score	Korean NHI: HBV DNA ≥20,000 IU/mL and ALT ≥80 IU/L EASL: HBV DNA ≥2000 IU/mL and ALT ≥33 IU/L in men and ≥25 IU/L in women New extended indication: HBV DNA ≥2000 IU/mL and any ALT	Total 5-year HCC risk: ○ Korean NHI: 2.5% ○ EASL: 2.1% ○ New extended indication: 1.1%
Lim 2021 [82]	Virtual CHB cohort based on Korean data Total HBsAg-positive population (2016): 1,478,500 Total diagnosed (2020): 1,125,400 Newly diagnosed: 19,600 On treatment (2019): 262,000	Base case: current eligibility requirements maintained through to 2035 Treat 70% + cirrhosis: treat 70% of eligible patients if current guidelines extended to include all cirrhotic patients Treat 70% + ULN: treat 70% of eligible patients if current guidelines lowered ALT restriction to ULN Treat 70% + ≥2000 IU/mL: treat 70% of eligible patients if current guidelines removed HBeAg and ALT restrictions and included all those with HBV DNA ≥2000 IU/mL	Disease burden outputs by 2035 compared with base case: ○ Treat 70% + cirrhosis: ↓ 4300 decompensated cirrhosis cases, ↓ 13,000 HCC cases, ↓ 11,800 deaths ○ Treat 70% + ULN: ↓ 7200 decompensated cirrhosis cases, ↓ 26,700 HCC cases, ↓23,000 deaths ○ Treat 70% + ≥2000 IU/mL: ↓ 9800 decompensated cirrhosis cases, ↓ 43,300 HCC cases, ↓ 37,000 deaths All scenarios were highly cost effective
Sanai 2020 [83]	CHB patients in Saudi Arabia using estimated national prevalence in 2017 HBsAg prevalence: 1.7% HCC incidence: 1500 cases 77% of patients aged 35–60 years	Base case: current scenario in Saudi Arabia Achieve WHO target: diagnose 90% of infections and treat 80% of high viral load patients by 2030 Diagnose and treat all: diagnose and treat all infected patients by 2022	Achieve WHO target strategy would cause 30% reduction in HCC and 35% reduction in liver-related deaths and would achieve positive ROI by 2021 Diagnose-and-treat-all strategy would cause 50% reduction in HCC and 55% reduction in liver-related deaths and would require ≥50% reduction in treatment costs to achieve positive ROI by 2028

ALT, alanine aminotransferase; CHB, chronic hepatitis B; EASL, European Association for the Study of the Liver; HBeAg, hepatitis B envelope antigen; HBsAg, hepatitis B surface antigen; HBV, hepatitis B virus; HCC, hepatocellular carcinoma; ICER, incremental cost-effectiveness ratio; IT, immune tolerant; NA, nucleos(t)ide analog; NHI, national health insurance; ROI, return on investment; ULN, upper limit of normal; WHO, World Health Organization.

## 7. Potential Strategies for Expanded HBV Treatment

Current international CHB guidelines are complicated and only recommend antiviral treatment in subsets of patients based on liver damage, serum HBV DNA, and ALT levels (Table 1) [3,4,6]. This is in contrast to hepatitis C or HIV where recent guidelines have promoted the early treatment of all infected patients regardless of end-organ damage [70]. However, evidence from multiple studies, as discussed in this review, demonstrates that patients currently ineligible for antiviral treatment may have liver damage and remain at risk for disease progression and HCC. Consequently, we believe that it is time to consider the adoption of expanded treatment strategies to reduce these risks.

As above-mentioned, the Chinese hepatology and infectious disease societies have recently adopted such an expanded treatment strategy [75]. In their latest guidelines, antiviral treatment is recommended in all HBsAg-positive patients with detectable serum HBV DNA (i.e., HBV DNA > 10–20 IU/mL) over the age of 30 years, regardless of ALT level. Under this strategy, 92% of all patients with HBV in China (75 million individuals) are now considered eligible for treatment. Antiviral treatment is also recommended for HBV DNA seropositive patients younger than 30 years of age with persistently elevated ALT (i.e., >upper limit of normal), those with compensated cirrhosis, and those with a risk factor for disease progression (≥grade 2 inflammation or ≥stage 2 fibrosis, a family history of HBV-related cirrhosis or HCC, or HBV-related extrahepatic manifestations).

**Figure 4:**
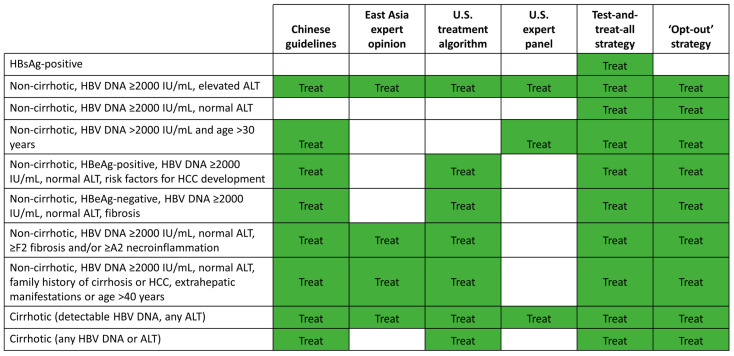
Potential expanded HBV treatment strategies. This figure summarizes the categories of CHB patients who would be eligible for antiviral treatment initiation under the proposed alternative treatment strategies. The Chinese guidelines [75], East Asian expert opinion [61], U.S. treatment algorithm [84] and U.S. expert panel [85] recommend initiation of antiviral treatment in more CHB patients compared with the current EASL, APASL, or AASLD guidelines, but include different patient subpopulations. The test-and-treat-all strategy would initiate antiviral treatment in all HBsAg-positive patients. The ‘opt-out’ strategy would initiate antiviral treatment in all cirrhotic patients and non-cirrhotic adult patients with HBV DNA ≥2000 IU/mL regardless of HBeAg and ALT status. AASLD, American Association for the Study of Liver Diseases; ALT, alanine aminotransferase; APASL, Asian Pacific Association for the Study of the Liver; CHB, chronic hepatitis B; EASL, European Association for the Study of the Liver; HBeAg, hepatitis B envelope antigen; HBsAg, hepatitis B surface antigen; HBV, hepatitis B virus; HCC, hepatocellular carcinoma.

There are several other expanded treatment strategies proposed throughout the world including an East Asia expert opinion [61], a U.S. treatment algorithm [84], a test-and-treat-all HBsAg-positive patients strategy, and expert recommendations for the simplification of current guidelines [85] (Figure 4). Both the East Asia expert opinion and the U.S. treatment algorithm propose initiating antiviral treatment in more CHB patients at risk of HCC and adverse liver outcomes than current EASL, AASLD, and APASL treatment guidelines [3,4,6,61,84]. However, they still require the measurement of HBV DNA and ALT levels, and fibrosis assessment prior to treatment decision-making. In contrast, the test-and-treat-all strategy, where all HBsAg-positive patients would be eligible for NA treatment, does not require HBV DNA testing and could reduce the diagnosis costs. However, this approach would require mass screening to diagnose all HBsAg-positive patients, which has major implications for public health policies. A test-and-treat-all protocol for HBV elimination has been successfully implemented in a national program in Uzbekistan [86].

The data that we reviewed collectively provide support for the simplification of treatment initiation strategies that incorporate broader treatment of adult patients with HBV DNA ≥2000 IU/mL, regardless of ALT levels. Furthermore, an ‘opt-out’ strategy to treat all non-cirrhotic patients with HBV DNA ≥2000 IU/mL, regardless of ALT levels, would first define the patients who may remain untreated with minimal long-term risk of disease progression and HCC, namely true ‘inactive carriers’. Compared with traditional guideline recommendations, this strategy would incorporate the treatment of (1) all cirrhotic patients, (2) gray-zone patients with viremia and normal ALT, and (3) IT patients. We submit that the data we have compiled in the preceding sections provide convincing evidence that the benefits of an expanded approach outweigh the costs and any risks associated with early treatment. This simplified approach would only require testing once for HBsAg status and HBV DNA levels, and HBV DNA testing could be omitted in regions with limited access. Optimal CHB treatment may also differ by country or region based on HBV prevalence, the costs of diagnostic testing and NA treatment, and reimbursement policies. An economic impact analysis of HBV in 25 countries suggested that an ‘opt-out’ strategy diagnosing 90% of infections and treating 80% of infected patients would be cost effective or cost saving in all countries [87].

The potential safety implications of expanding the initiation of long-term antiviral treatment should, of course, be considered. However, accumulating clinical experience suggests a minimal risk of side effects associated with current antiviral treatment options, and this must therefore be balanced against the risk of disease progression and HCC in untreated CHB patients. Other potential concerns related to long-term antiviral treatment are resistance and poor adherence. ETV, TDF, and TAF have a high barrier to resistance; no resistance to TDF or TAF has been detected, and resistance to ETV is rare among treatment-naïve patients [3,4]. Furthermore, adherence rates to NA therapies are generally high [39]. Finally, and importantly, the current research to achieve a functional cure for CHB makes us optimistic that any concerns related to long-term NA therapy are likely to be time limited. Although HBV cure may not be anticipated in the immediate future, the bar to initiate antiviral treatment may certainly be lowered.

In conclusion, the available molecular, clinical, and economic data provide a strong rationale for the earlier initiation of antiviral treatment in CHB patients to reduce the risk of disease progression and HCC. Adoption of such a simplified ‘treat to prevent’ approach could save countless lives in a cost-effective manner. In parallel, investment in research efforts into finding a functional cure for CHB should continue to dramatically change the treatment paradigm in the future.

## Figures and Tables

**Table 1:** Antiviral treatment criteria in the international guidelines.

	EASL 2017 [3]	AASLD 2018 [6]	APASL 2016 [4]
**No cirrhosis**	HBeAg-positive/negative AND HBV DNA > 2000 IU/mL AND ALT > ULN * and/or moderate liver necroinflammation or fibrosis ^†^	HBeAg-positive AND HBV DNA > 20,000 IU/mL AND ALT ≥ 2× ULN *	HBeAg-positive AND HBV DNA > 20,000 IU/mL AND ALT > 2× ULN *
	HBV DNA > 20,000 IU/mL AND ALT > 2× ULN *	HBeAg-negative AND HBV DNA ≥2000 IU/mL AND ALT ≥ 2× ULN *	HBeAg-negative AND HBV DNA > 2000 IU/mL AND ALT > 2× ULN *
	HBeAg-positive/negative AND family history of HCC or cirrhosis and extrahepatic manifestations	HBV DNA ≥2000 IU/mL AND ALT > ULN * AND significant necroinflammation or fibrosis ^‡^ or age > 40 years	Any HBV DNA or ALT if moderate to severe inflammation or significant fibrosis
**Cirrhosis**	Detectable HBV DNA Any ALT level	HBV DNA < 2000 IU/mL ^§^ Any ALT level	HBV DNA > 2000 IU/mL ^§^ Any ALT level

Please note that the national treatment guidelines may differ from international treatment guidelines. * The EASL and APASL recommended ULN is 40 IU/L, the AASLD recommended ULN is 35 U/L for males and 25 U/L for females; ^†^ Patients with HBeAg-positive chronic infection may be treated if older than 30 years regardless of the severity of liver histology; ^‡^ ALT > ULN and significant necroinflammation or fibrosis, but HBV DNA < 2000 IU/mL, treat; ^§^ For compensated cirrhosis. Patients with decompensated cirrhosis should be treated if there is detectable HBV DNA with any ALT levels. AASLD, American Association for the Study of Liver Diseases; ALT, alanine aminotransferase; APASL, Asian Pacific Association for the Study of the Liver; EASL, European Association for the Study of the Liver; HBeAg, hepatitis B e-antigen; HBV, hepatitis B virus; HCC, hepatocellular carcinoma; ULN, upper limit of normal.

## Data Availability

No new data were created or analyzed in this study. Data sharing is not applicable to this article.

## Supplementary Materials

The following supporting information can be downloaded at: https://www.mdpi.com/article/10.3390/v15040997/s1, Figure S1: CHB disease phases; Figure S2: Flow diagram of literature search for clinical review; Table S1: Risk of liver damage in CHB patients with normal or minimally elevated ALT levels; File S1: Phases of CHB disease; File S2: Search strategy for clinical review; File S3: Additional literature.
