# Supplementary material for: Evidence for Benefits of Early Treatment Initiation for Chronic Hepatitis B
Source: Viruses. 2023 Apr 18;15(4):997. doi: 10.3390/v15040997 (PMC10142077; doi:10.3390/v15040997)
Supplement: Supplementary file 1 [file viruses-15-00997-s001.zip › viruses-2343113-supplementary.pdf]

Supplementary File 1: Phases of CHB disease

Chronic hepatitis B (CHB) infection is divided into four disease phases that are closely associated with age but are not necessarily sequential (Supplementary Figure S1): hepatitis B envelope antigen (HBeAg)-positive chronic infection (also known as immune tolerant [IT]), HBeAg-positive chronic hepatitis (also known as immune active [IA]), HBeAg-negative chronic infection (also known as immune control [IC], immune clearance, or immune inactive), and HBeAg-negative chronic hepatitis (also known as immune escape).[1] Some patients will not fall into any of these disease phases and are described as belonging to the 'grey zone',[2] which includes adult HBeAg-positive patients with normal alanine aminotransferase (ALT) but high or decreasing HBV DNA levels and adult HBeAg-negative patients with normal ALT but increasing HBV DNA levels.

Supplementary File 2: Search strategy for clinical review

PubMed searches were performed for HBV and adverse liver outcomes, HBV integration, and HBV treatment and cost (Supplementary Figure S2).

PubMed search terms:

- HBV and adverse liver outcomes: "hepatitis B AND risk AND predict OR inform AND liver cirrhosis OR liver fibrosis OR hepatocellular carcinoma OR liver neoplasms OR disease progression AND clinical OR cohort OR registry OR study OR trial OR prospective OR retrospective"
- HBV integration: "hepatitis B virus OR HBV AND integration OR translocation AND hepatocellular carcinoma OR liver cancer OR liver neoplasms" and "hepatitis B virus OR HBV AND hepatocellular carcinoma OR liver cancer OR liver neoplasms AND molecular mechanisms"
- HBV treatment and cost: "hepatitis B AND chronic AND treatment and cost"

Inclusion criteria: articles of any type published up to 10 June 2021 were included.

Abstracts from three major international liver congresses (The International Liver Congress, The Liver Meeting and The Conference of the Asian Pacific Association for the Study of the Liver [APASL]) from 2019–2021 were also searched using the term ‘HBV’. Abstracts were manually reviewed and search results graded according to evidence and analysis type, strength of conclusions, and relevance to search objective. All authors reviewed results of the literature search and provided feedback as to which publications to omit and additional articles to include that had not been identified in the literature search.

For completeness, a full list of relevant literature – including additional papers suggested by authors during review of the selected literature – is provided in Supplementary File 2.

Supplementary Figure S1. CHB disease phases

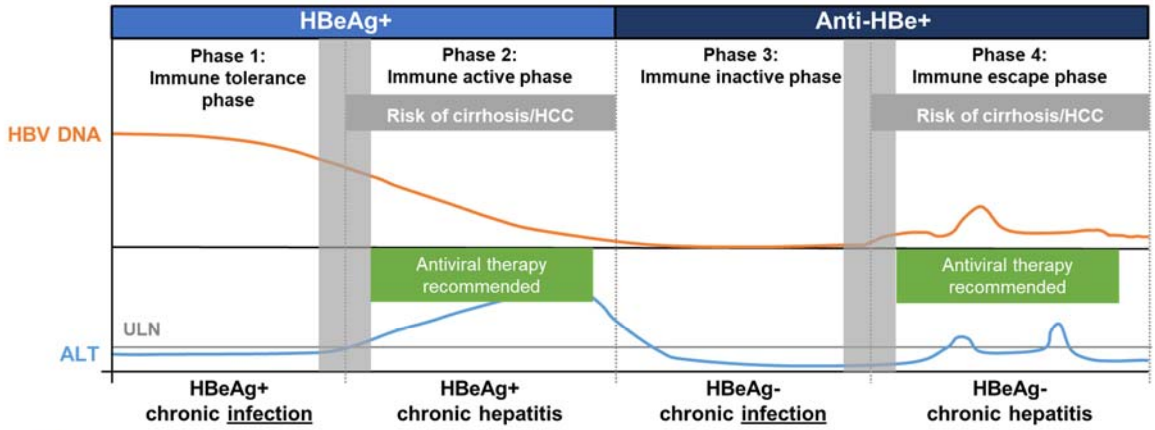

HBV DNA	$>10^7$ IU/mL (EASL)<br>$>10^6$ IU/mL (AASLD)	10^4–10^7 IU/mL (EASL)<br>>20,000 IU/mL (AASLD)	<2000 IU/mL*	>2000 IU/mL
ALT	Persistently normal (EASL)<br>Normal or minimally elevated (AASLD)	Elevated	Normal	Persistently or fluctuating elevated
Fibrosis	Minimal or no necroinflammation or fibrosis	Moderate or severe necroinflammation<br>Progression of fibrosis (EASL)<br>With or without fibrosis (AASLD)	Absence of significant necroinflammation and variable levels of fibrosis (AASLD)	Necroinflammation and fibrosis (EASL)<br>Moderate or severe necroinflammation with or without fibrosis (AASLD)

*Some patients may have HBV DNA >2000 IU/mL, persistently normal ALT and minimal hepatic necroinflammatory activity and low fibrosis (EASL).

The upper panel summarizes the changes in HBeAg status and HBV DNA and ALT levels associated with the four CHB disease phases. The lower panel includes the definitions of these phases according to EASL and AASLD guidelines.[1,3] Patients in the HBeAg-positive chronic infection/immune- tolerant disease phase are HBeAg positive, with high HBV DNA levels and normal or minimally elevated ALT. These patients are considered to have minimal or no necroinflammation or fibrosis and are typically ineligible for antiviral treatment. Patients in the HBeAg-positive chronic hepatitis/immune-active disease phase are HBeAg positive, with decreasing HBV DNA levels and elevated ALT. These patients have evidence of liver damage, are considered at risk of cirrhosis and/or HCC, and are therefore eligible for antiviral treatment. Patients in the HBeAg-negative chronic infection/immune-control phase are HBeAg negative, with low HBV DNA levels and normal ALT. These patients are considered to have no significant necroinflammation, may have variable levels of fibrosis and are typically ineligible for antiviral treatment. Patients in the HBeAg-negative chronic hepatitis/immune-escape disease phase are HBeAg negative, with HBV DNA above 2000 IU/mL and persistently or fluctuating elevated ALT. These patients have evidence of liver damage, are considered at risk of cirrhosis and/or HCC, and are therefore eligible for antiviral treatment. AASLD, American Association for the Study of

Liver Diseases; ALT, alanine aminotransferase; CHB, chronic hepatitis B; EASL, European Association for the Study of the Liver; HBe, hepatitis B e; HBeAg, hepatitis B envelope antigen; HBV, hepatitis B virus; HCC, hepatocellular carcinoma; ULN, upper limit of normal. (Figure adapted with permission from ©NICE 2013 Hepatitis B (chronic): diagnosis and management clinical guidelines. Available from https://www.nice.org.uk/guidance/cg165. All rights reserved. NICE guidance is prepared for the National Health Service in England. All NICE guidance is subject to regular review and may be updated or withdrawn. NICE accepts no responsibility for the use of its content in this publication.)

Supplementary Figure S2. Flow diagram of literature search for clinical review

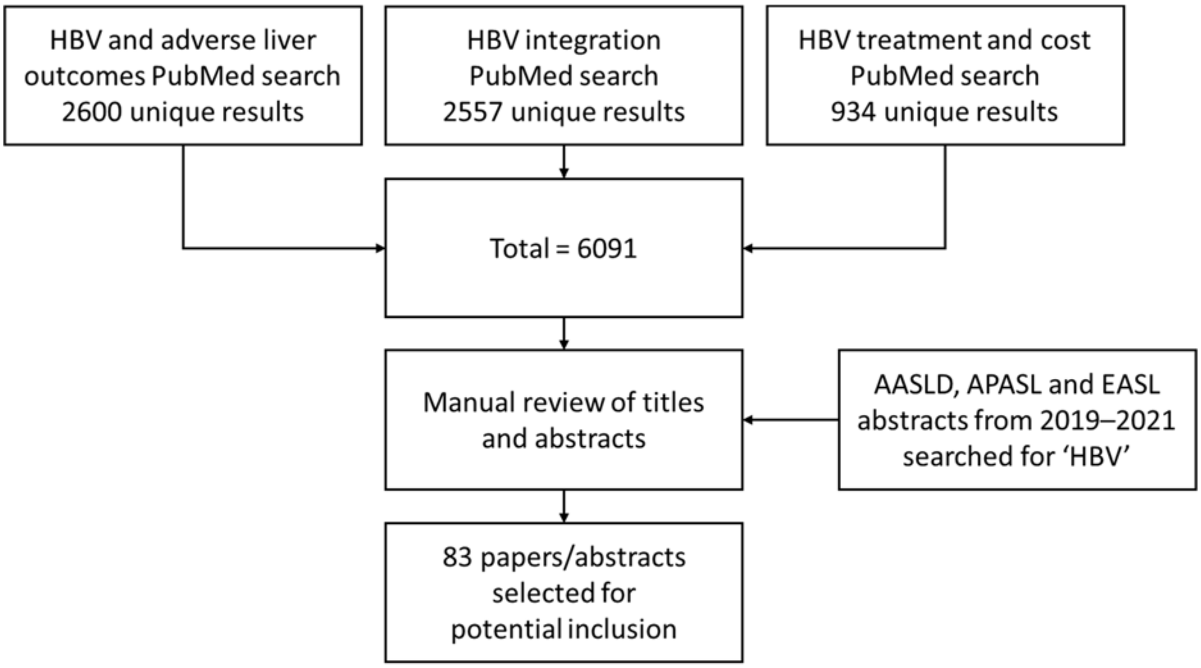

This flow diagram summarizes the literature search performed as part of this clinical review. Three PubMed searches were performed for HBV and adverse liver outcomes, HBV integration, and HBV treatment and cost. Articles of any type published up to 10 June 2021 were included, leading to a total of 6091 papers. Abstracts from three major international liver congresses (The International Liver Congress, The Liver Meeting, and The Conference of the Asian Pacific Association for the Study of the Liver [APASL]) from 2019–2021 were also searched using the term 'HBV'. All abstracts were manually reviewed and search results graded according to evidence and analysis type, strength of conclusions, and relevance to search objective. Eighty-three papers and congress abstracts were selected for potential inclusion in this clinical review, but only those directly relevant to the topic were cited due to space constraints. AASLD, American Association for the Study of Liver Diseases; EASL, European Association for the Study of the Liver; HBV, hepatitis B virus.

Supplementary Table S1. Risk of liver damage in CHB patients with normal or minimally elevated ALT levels

Author and year	Study type	Patient population	Key finding
**HBeAg-positive IT disease phase**			
Lai 2007[4]	Retrospective review	CHB patients with persistently normal ALT, ALT 1–1.5x ULN*, and ALT >1.5x ULN* (N=192)	• 18% of patients with persistently normal ALT had stage 2+ fibrosis and 34% had grade 2 or 3 inflammation
Kumar 2008[5]	Prospective study	Asymptomatic CHB patients with persistently normal ALT or persistently or intermittently elevated ALT† (N=1387)	• Fibrosis stage ≥2 was present in 40% (HBeAg positive) and 13% (HBeAg negative) of CHB patients with persistently normal ALT
Wong 2008[6]	Prospective study	Treatment-naïve HBeAg-negative CHB patients stratified into five groups according to ALT level (N=1197)	• Patients with ALT levels >0.5–1x ULN‡ had significantly increased risk of possible and probable cirrhosis compared with patients with ALT levels ≤0.5x ULN ($p<0.001$)‡
Zhang 2021[7]	Meta-analysis	19 studies in treatment-naïve CHB patients with normal ALT* (N=2771)	• Approximately 1/3 patients showed significant histological changes and 3% had cirrhosis

*ULN defined as 40 U/L. †Persistently normal defined as ≤40 IU/L, persistently or intermittently elevated defined as >40 IU/L. ‡ULN defined as 58 IU/L. ALT, alanine aminotransferase; CHB, chronic hepatitis B; HBeAg, hepatitis B envelope antigen; IT, immune tolerant; ULN, upper limit of normal.

Supplementary references

1. European Association for the Study of the Liver (EASL). EASL 2017 Clinical Practice Guidelines on the management of hepatitis B virus infection. *J Hepatol*. 2017;67(2):370-398.

2. Seto WK, Lo YR, Pawlotsky JM, Yuen MF. Chronic hepatitis B virus infection. *Lancet*. 2018;392(10161):2313-2324.

3. Terrault NA, Lok ASF, McMahon BJ. Update on prevention, diagnosis, and treatment of chronic hepatitis B: AASLD 2018 hepatitis B guidance. *Hepatology*. 2018;67(4):1560-1599.

4. Lai M, Hyatt BJ, Nasser I, Curry M, Afdhal NH. The clinical significance of persistently normal ALT in chronic hepatitis B infection. *J Hepatol*. 2007;47(6):760-767.

5. Kumar M, Sarin SK, Hissar S. Virologic and histologic features of chronic hepatitis B virus-infected asymptomatic patients with persistently normal ALT. *Gastroenterology*. 2008;134(5):1376-1384.

6. Wong GLH, Wong VWS, Choi PCL. Evaluation of alanine transaminase and hepatitis B virus DNA to predict liver cirrhosis in hepatitis B e antigen-negative chronic hepatitis B using transient elastography. *Am J Gastroenterol*. 2008;103(12):3071-3081.

7. Zhang C, Li JW, Wu Z, Zhao H, Wang GQ. Significant histologic changes are not rare in treatment-naive hepatitis B patients with normal alanine aminotransferase level: a meta-analysis. *J Clin Transl Hepatol*. 2021;9:615-625.

Supplementary File 2: Additional literature

Studies evaluating HCC risk in CHB patients with no antiviral treatment

Liang LY, et al. Serum fibrosis index-based risk score predicts hepatocellular carcinoma in untreated patients with chronic hepatitis B. *Clin Mol Hepatol.* 2021;27:499-509.

Tran S, et al. Initial evaluation, long-term monitoring, and hepatocellular carcinoma surveillance of chronic hepatitis B in routine practice: a nationwide US study. *Am J Gastroenterol.* 2021;116:1885-1895.

Choi WM, et al. Association between pretreatment baseline viral load and on-treatment risk of hepatocellular carcinoma in chronic hepatitis B. *Hepatology*. 2020;72:A748.

Fan R, et al. aMAP risk score predicts hepatocellular carcinoma development in patients with chronic hepatitis*. J Hepatol.* 2020;73:1368-1378.

Jang H, et al. Revisiting the impact of HBeAg-status of antiviral treatment on risk of hepatocellular carcinoma development: a nationwide multicenter study. *Hepatol.* 2020;73;A808.

Lee HA, et al. Extremely low risk of hepatocellular carcinoma development in patients with chronic hepatitis B in immune-tolerant phase. *Aliment Pharmacol Ther.* 2020;52:196-204.

Seo SI, et al. Predictive factors for risk of hepatocellular carcinoma in immune inactive chronic hepatitis B. *Clin Res Hepatol Gastroenterol.* 2020;44:711-717.

Wong GL, et al. Machine learning models to predict hepatocellular carcinoma in patients with chronic viral hepatitis: a territory-wide study from hospital authority data collaboration lab (HADCL) in 2000-2017. *Hepatol.* 2020;72:A767.

Clutter DS, et al. Reduced risk of hepatocellular carcinoma among individuals with isolated anti-hepatitis B core antibody positivity compared with chronic hepatitis B infection in a large Northern California cohort. *Hepatol.* 2019;70:A975.

Zhang C, et al. Association between aspartate aminotransferase-to-platelet ratio index and hepatocellular carcinoma risk in patients with chronic hepatitis: a meta-analysis of cohort study. *Dis Markers.* 2019;2019:2046825.

Papatheodoridis GV, et al. The risk of hepatocellular carcinoma decreases after the first 5 years of entecavir or tenofovir in Causasians with chronic hepatitis B. *Hepatology*. 2017;66:1444-1453.

Tseng T-C, et al. Fibrosis-4 index helps identify HBV carriers with the lowest risk of hepatocellular carcinoma. *Am J Gastroenterol*. 2017;112:1564-1574.

Raffety E, Fattovich G and Donato F. Incidence of hepatocellular carcinoma in untreated subjects with chronic hepatitis B: a systematic review and meta-analysis. *Liver Int.* 2016;36:1239-1251.

Su T-H, et al. Four-year entecavir therapy reduces hepatocellular carcinoma cirrhotic events and mortality in chronic hepatitis B patients. *Liver Int*. 2016;36:1755-1764.

Yang H-I, et al. Incorporating serum level of hepatitis B surface antigen or omitting level of hepatitis B virus DNA does not affect calculation of risk for hepatocellular carcinoma in patients without cirrhosis. *Clin Gastroenterol Hepatol.* 2016;14:461-468.e2.

Liu J, et al. Spontaneous seroclearance of hepatitis B seromarkers and subsequent risk of hepatocellular carcinoma. *Gut*. 2014;63:1648-1657.

Thiele M, et al. Large variations in risk of hepatocellular carcinoma and mortality in treatment naïve hepatitis B patients: systematic review with meta-analyses. *PLoS One.* 2014;9:e107177.

Wong G L-H, et al. Liver stiffness-based optimization of hepatocellular carcinoma risk score in patients with chronic hepatitis B. *J Hepatol*. 2014;60:339-345.

Wu CY, et al. Association of nucleos(t)ide analogue therapy with reduced risk of hepatocellular carcinoma in patients with chronic hepatitis B – a nationwide cohort study. *Gastroenterology*. 2014;147:143-51.e5.

Hosaka T, et al. Long-term entecavir treatment reduced hepatocellular carcinoma incidence in patients with hepatitis B virus infection. *Hepatology*. 2013;58:98-107.

Izzo F, et al. Prospective screening increases the detection of potentially curable hepatocellular carcinoma: results in 8,900 high-risk patients. *HPB (Oxford).* 2013;15:985-990.

Lin Y-J, et al. Predictability of liver-related seromarkers for the risk of hepatocellular carcinoma in chronic hepatitis B patients. *PLoS One*. 2013;8:e61448.

Wen CP, et al. Hepatocellular carcinoma risk prediction model for the general population: the predictive power of transaminases. *J Natl Cancer Inst*. 2012;104:1599-1611.

Liao Y, et al. Precore mutation of hepatitis B virus may contribute to hepatocellular carcinoma risk: evidence from an updated meta-analysis. *PLoS One*. 2012;7:e38394.

Chen C-F, et al. Changes in serum levels of HBV DNA and alanine aminotransferase determine risk for hepatocellular carcinoma. *Gastroenterology.* 2011;141:1240-1248.

Yang H-I, et al. Risk estimation for hepatocellular carcinoma in chronic hepatitis B (REACH-B): development and validation of a predictive score. *Lancet Oncol*. 2011;12:568-574.

Chen J-D, et al. Carriers of inactive hepatitis B virus are still at risk for hepatocellular carcinoma and liver-related death. *Gastroenterology.* 2010;138:1747-1754.

Chen C-J, et al. Risk of hepatocellular carcinoma across a biological gradient of serum hepatitis B virus DNA level. *JAMA.* 2006;295:65-73.

Studies evaluating risk of other adverse liver outcomes in patients with no antiviral treatment

Chang X, et al. A novel nomogram to predict evident histological liver injury in patients with HBeAg-positive chronic hepatitis B virus infection. *EBioMedicine*. 2021;67:103389.

Li M, et al. Correlation among age, sex, and liver diseases-related mortality risk in patients with hepatitis B virus-related liver cirrhosis. *Zhonghua Gan Zang Bing Za Zhi.* 2021;29:403-408. [Article in Chinese].

Graf C, et al. Clinical outcomes in untreated European patients with chronic hepatitis B virus infection after seven years: a prospective longitudinal study (Albatros study). *Hepatol.* 2019;70:A654.

Shim J-J, et al. Serum alanine aminotransferase level and liver-related mortality in patients with chronic hepatitis B: a large national cohort study. *Liver Int*. 2018;38:1751-1759.

Sun Y, et al. Neglected significant fibrosis in young HBV patients: evidence from China registry of hepatitis B. *Hepatol Int.* 2016;10:S238.

Qu LS, et al. Effect of serum hepatitis B surface antigen levels on predicting the clinical outcomes of chronic hepatitis B infection: a meta-analysis. *Hepatol Res*. 2015;45:1004-1013.

Chien Y-C, et al. Incomplete hepatitis B immunization, maternal carrier status, and increased risk of liver diseases: a 20-year cohort study of 3.8 million vaccinees. *Hepatology*. 2014;60:125-132.

Post SE, et al. A simulation shows that early treatment of chronic hepatitis B infection can cut deaths and be cost-effective. *Health Aff (Millwood).* 2011;30:340-348.

Wong G L-H, et al. Evaluation of alanine transaminase and hepatitis B virus DNA to predict liver cirrhosis in hepatitis B e antigen-negative chronic hepatitis B using transient elastography. *Am J Gastroenterol*. 2008;103:3071-3081.

Iloeje UH, et al. Risk and predictors of mortality associated with chronic hepatitis B infection. *Clin Gastroenterol Hepatol*. 2007;5:921-931.

Iloeje UH, et al. Predicting cirrhosis risk based on the level of circulating hepatitis B viral load. *Gastroenterology*. 2006;130:678-686.

Studies evaluating HBV and HCC/adverse liver outcomes in CHB patients on antiviral treatment

Chang T-S, et al. Long-term risk of primary liver cancers in entecavir versus tenofovir treatment for chronic hepatitis B. *Sci Rep*. 2021;11:1365.

Chon HY, et al. Impact of antiviral therapy on risk prediction model for hepatocellular carcinoma development in patients with chronic hepatitis B. *Hepatol Res*. 2021;51:406-416.

Farina et al. Endoscopic surveillance of esophageal varices could be refined in HBV compensated cirrhotics taking tenofovir or entecavir: an 11-year real-life study. *J Hepatol.* 2021;75(Suppl2):S191-S865.

Hsu Y-C, et al. Once-daily tenofovir disoproxil fumarate in treatment-naïve Taiwanese patients with chronic hepatitis B and minimally raised alanine aminotransferase (TORCH-B): a multicentre, double-blind, placebo-controlled, parallel-group, randomised trial. *Lancet Infect Dis*. 2021;21:823-833.

Nam H, et al. Prediction of hepatocellular carcinoma by on-therapy response of noninvasive fibrosis markers in chronic hepatitis B. *Am J Gastroenterol.* 2021;116:1657-1666.

Papatheodoridis GV, et al. Predictive performance of newer Asian hepatocellular carcinoma risk scores in treated Caucasians with chronic hepatitis B. *JHEP Rep*. 2021;3:100290.

Lee HW, et al. An optimized hepatocellular carcinoma prediction model for chronic hepatitis B with well-controlled viremia. *Liver Int*. 2020;40:1736-1743.

Marcellin P, et al. Final analysis of the international observational S-Collate study of peginterferon alfa-2a in patients with chronic hepatitis B. *PLoS One.* 2020;15:e0230893.

Simon TG, et al. Association of aspirin with hepatocellular carcinoma and liver-related mortality. *N Engl J Med.* 2020;382:1018-1028.

Yang H-I, et al. Real-world effectiveness from the Asia Pacific Rim Liver Consortium for HBV risk score for the prediction of hepatocellular carcinoma in chronic hepatitis B patients treated with oral antiviral therapy. *J Infect Dis.* 2020;221:389-399.

Yip TC-F, et al. Reassessing the accuracy of PAGE-B-related scores to predict hepatocellular carcinoma development in patients with chronic hepatitis B. *J Hepatol*. 2020;72:847-854.

Yip TC-F, et al. Evaluation of page-b and modified page-b scores to guide hepatocellular carcinoma surveillance in chronic hepatitis B patients on antiviral therapy – a territory-wide study of 32,150 subjects. *J Hepatol.* 2020;73: S599-S600.

Chon H-Y, et al. Dynamics of a transient elastography-based risk prediction model for hepatocellular carcinoma treated with antiviral therapy in chronic hepatitis B: a multi-center retrospective cohort study from the Korean Transient Elastography Study Group. *J Hepatol.* 2019;70:e255-e256.

Desalegn H, et al. Predictors of mortality in patients under treatment for chronic hepatitis B in Ethiopia: a prospective cohort study. *BMC Gastroenterol*. 2019;19:74.

Kwon JH, et al. Low risk of liver disease progression in untreated immune tolerant phase chronic hepatitis B patients compared to treated immune active phase patients; multi-center cohort study up to 15 years. *Hepatol.* 2019;70:A937.

Marzano A, et al. Liver stiffness-based model for portal hypertension and hepatocellular cancer risk in HBV responsive to antivirals. *Minerva Gastroenterol Dietol*. 2019;65:11-19.

Hsu Y-C, et al. Development of a scoring system to predict hepatocellular carcinoma in Asians on antivirals for chronic hepatitis B. *J Hepatol*. 2018;69:278-285.

Huang JP, et al. Impact of the timing of hepatitis B virus identification and anti-hepatitis B virus therapy initiation on the risk of adverse liver outcomes for patients receiving cancer therapy. *Cancer*. 2017;123:3367-3376.

Li Z-Q, et al. The development of hepatocarcinoma after long-term antivirus treatment of Chinese patients with chronic hepatitis B virus infection: incidence, long-term outcomes and predictive factors. *Clin Res Hepatol Gastroenterol*. 2017;41:311-318.

Lok AS, et al. Antiviral therapy for chronic hepatitis B viral infection in adults: a systematic review and meta-analysis. *Hepatology*. 2016;63:284-306.

Papatheodoridis G, et al. PAGE-B predicts the risk of developing hepatocellular carcinoma in Caucasians with chronic hepatitis B on 5-year antiviral therapy. *J Hepatol.* 2016;64:800-806.

Shim J-J, et al. Predictive value of antiviral effects in the development of hepatocellular carcinoma in the general Korean population with chronic hepatitis B. *Gut Liver*. 2016;10:962-968.

Kim WR, et al. Impact of long-term tenofovir disoproxil fumarate on incidence of hepatocellular carcinoma in patients with chronic hepatitis B. *Cancer*. 2015;121:3631-3638.

Lampertico P, et al. The long-term benefits of nucleos(t)ide analogs in compensated HBV cirrhotic patients with no or small esophageal varices: a 12-year prospective cohort study. *J Hepatol*. 2015;63:1118-1125.

Yin J, et al. Hepatitis B virus combo mutations improve the prediction and active prophylaxis of hepatocellular carcinoma: a clinic-based cohort study. *Cancer Prev Res (Phila)*. 2015;8:978-988.

Arends P, et al. Prediction of hepatocellular carcinoma in entecavir treated patients: results from 744 chronic hepatitis B patients in a European multicenter study (VIRGIL). *Hepatol.* 2013;58:A949.

Wong GL-H, et al. Entecavir treatment reduces hepatic events and deaths in chronic hepatitis B patients with liver cirrhosis. *Hepatology*. 2013;58:1537-1547.

Studies evaluating HCC risk in CHB patients outside current treatment guidelines

Kim G-A, et al. Moderate levels of serum hepatitis B virus DNA are associated with the highest risk of hepatocellular carcinoma in chronic hepatitis B patients. *Aliment Pharmacol Ther*. 2020;51:1169-1179.

Choi GH, et al. High risk of clinical events in untreated HBeAg-negative chronic hepatitis B patients with high viral load and no significant ALT elevation. *Aliment Pharmacol Ther*. 2019;50:215-226.

Sinn DH, et al. The risk of hepatocellular carcinoma among chronic hepatitis B virus-infected patients outside current treatment criteria. *J Viral Hepat*. 2019;26:1465-1472.

Kim G-A, et al. High risk of hepatocellular carcinoma and death in patients with immune-tolerant-phase chronic hepatitis B. *Gut*. 2018;67:945-952.

Tseng T-C, et al. HCC risk stratified by HBcrAg in chronic hepatitis B patients in grey zone of antiviral treatment. GHS. Oral 278.

Alshuwaykh O, et al. The incidence of hepatocellular carcinoma in chronic hepatitis B virus infection subjects not meeting criteria for antiviral therapy. *Hepatology.* 2020;72:A776.

Kim G-A, et al. Treatment recommendations for chronic hepatitis B and the risk of hepatocellular carcinoma. *J Hepatol.* 2019(Suppl);70:e462.

Seong G, et al. Risk of hepatocellular carcinoma in patients with immune-tolerant hepatitis B. *J Hepatol.* 2019(Suppl);70:e266.

Studies evaluating adverse liver outcome risk in CHB patients outside current treatment guidelines

Zhang C, et al. Significant histologic changes are not rare in treatment-naïve hepatitis B patients with normal alanine aminotransferase level: a meta-analysis. *J Clin Transl Hepatol*. 2021;9:615-625.

Kumar M, et al. Virologic and histologic features of chronic hepatitis B virus-infected asymptomatic patients with persistently normal ALT. *Gastroenterology*. 2008;134:1376-1384.

Lai M, et al. The clinical significance of persistently normal ALT in chronic hepatitis B infection. *J Hepatol*. 2007;47:760-767.

Wang C-Y, et al. Nomogram for predicting significant liver injury in chronic hepatitis B patients with immune tolerance. *J Hepatol*. 2021;75(Suppl 2): S191-S865

Wang Y, et al. Histologic fibrosis across the natural course of chronic hepatitis B virus infection: a systemic review and meta-analysis. *Hepatol Int.* 2020;14:S30.

Jeon MY, et al. Significant lower risk of liver-related events development in chronic hepatitis B patients with immune tolerant phase than those with antiviral therapy. *Hepatology.* 2019:70:A681.

Zeng D-W, et al. Value of hepatitis B surface antigen in the prediction of liver injury among patients with immune-tolerant phase chronic hepatitis B. *Hepatology.* 2019:70:A1001.

Chan HLY, et al. Effects of tenofovir disoproxil fumarate in hepatitis B e antigen-positive patients with normal levels of alanine aminotransferase and high levels of hepatitis B virus DNA. *Gastroenterology*. 2014;146:1240-1248.

Other studies evaluating HBV and liver outcomes

Poynard T, et al. Staging chronic hepatitis B into seven categories, defining inactive carriers and assessing treatment impact using a fibrosis biomarker (FibroTest®) and elastography (FibroScan®). *J Hepatol*. 2014;61:994-1003.

Guichard C, et al. Integrated analysis of somatic mutations and focal copy-number changes identifies key genes and pathways in hepatocellular carcinoma. *Nat Genet*. 2012;44:694-698.

Literature relating to mechanistic rationale for early HBV treatment

Erken, et al. Quantified integrated hepatitis B virus DNA is related to viral activity in chronic hepatitis B patients. *J Hepatol.* 2021;75(Suppl2):S191-S865.

Hu G, et al. Liver damage favors the eliminations of HBV integration and clonal hepatocytes in chronic hepatitis B. *Hepatol Int*. 2021;15:60-70.

Ramirez R, et al. Transcriptome analyses reveal variation of liver inflammation across phases of chronic hepatitis B infection. *J Hepatol.* 2021;75(Suppl2):S1716.

Svicher V, et al. Whole exome HBV DNA integration is independent of the intrahepatic HBV reservoir in HBeAg-negative chronic hepatitis B. *Gut*. 2021;70:2337-2348.

Zheng B, et al. The landscape of cell-free HBV integrations and mutations in cirrhosis and hepatocellular carcinoma patients. *Clin Cancer Res.* 2021;27:3772-3783.

Chow N, et al. Long term nucleos(t)ide analogue therapy reduced the extent of HBV DNA integration in chronic hepatitis B patients. *Hepatology.*2020;72:A22.

Hsu Y-C, et al. Tenofovir disoproxil fumarate treatment reduces the number of transcriptionally active viral integrations in chronically infected HBV patients. *Hepatology.*2020;72:A16.

Asahina Y, et al. Gene mutational profile and viral integration in hepatocellular carcinoma with or without HBV/HCV suppression. *Hepatol.* 2019;70.

Asahina Y, et al. Comprehensive analysis of cancer gene mutations and viral integration in hepatocellular carcinoma arising from non-fibrotic liver. *Hepatol.* 2019;70:A114.

Hsia K, et al. Viral integration in genome of hepatocellular carcinoma. *Mod Pathol.* 2019;32(Suppl 2):22.

Tatsuno K, et al. Impact of AAV2 and hepatitis B virus integration into genome on development of hepatocellular carcinoma in patients with prior hepatitis B virus infection. *Clin Cancer Res*. 2019;25:6217-6227.

Duan M, et al. Diverse modes of clonal evolution in HBV-related hepatocellular carcinoma revealed by single-cell genome sequencing. *Cell Res.* 2018;28:359-373.

Kuwai-Kitahata F, et al. Comprehensive analyses of mutations and hepatitis B virus integration in hepatocellular carcinoma with clinicopathological features. *J Gastroenterol*. 2016;51:473-486.

Larsson SB, et al. Integration of hepatitis B virus DNA in chronically infected patients assessed by Alu-PCR. *J Med Virol*. 2018;90:1568-1575.

Mason WS, et al. HBV DNA integration and clonal hepatocyte expansion in chronic hepatitis B patients considered immune tolerant. *Gastroenterology*. 2016;151:986-98.e4.

Park J-J, et al. Hepatitis B virus-specific and global T-cell dysfunction in chronic hepatitis B. *Gastroenterology*. 2016;150:684-95.e5.

Zhao LH, et al. Genomic and oncogenic preference of HBV integration in hepatocellular carcinoma. *Nat Commun*. 2016;7:12992.

Tu T, et al. Clonal expansion of hepatocytes with a selective advantage occurs during all stages of chronic hepatitis B virus infection. *J Viral Hepat*. 2015;22:737-753.

Mukherjee RM, et al. Reduced expression of DNA damage repair genes high mobility group box1 and poly(ADP-ribose) polymerase1 in inactive carriers of hepatitis B virus infection – a possible stage of viral integration. *J Clin Exp Hepatol*. 2013;3:89-95.

Kennedy PTF, et al. Preserved T-cell function in children and young adults with immune-tolerant chronic hepatitis B. *Gastroenterology*. 2012;143:637-645.

Wang H-Y, et al. Distinct hepatitis B virus dynamics in the immunotolerant and early immunoclearance phases. *J Virol*. 2010;84:3454-3463.

Studies evaluating HCC and other adverse liver outcome risk in HBV +/- HIV-infected patients

Wandeler G, et al. Incidence of hepatocellular carcinoma in HIV/HBV-coinfected patients on tenofovir therapy: relevance for screening strategies. *J Hepatol*. 2019;71:274-280.

Marcon PDS, et al. Incidence of hepatocellular carcinoma in patients with chronic liver disease due to hepatitis B or C and coinfected with the human immunodeficiency virus: a retrospective cohort study. *World J Gastroenterol*. 2018;24:613-622.

Naggie S, et al. Risk of HCC in patients with HBV infection with and without HIV coinfection: a US administrative claims analysis. *Hepatology.* 2020;72:A809.

Grant JL, et al. Changes in liver stiffness after ART initiation in HIV-infected Nigerian adults with and without chronic HBV. *J Antimicrob Chemother*. 2019;74:2003-2008.

Liu G, et al. HIV infection and the risk of hepatocellular carcinoma in patients with hepatitis B Virus co-infection: a propensity score-matched study. *Open Forum Infect Dis.* 2019;6(Suppl 2):S188.

Vinikoor MJ, et al. Impact of antiviral therapy on liver fibrosis among human immunodeficiency virus-infected adults with and without HBV co-infection in Zambia. *Clin Infect Dis.* 2017;64:1343-1349.

Literature evaluating cost effectiveness of CHB treatment strategies

Zhang S, et al. Cost-effectiveness of expanded antiviral treatment for chronic hepatitis B virus infection in China: an economic evaluation. *In Press.*

Toy M, et al. Cost-effectiveness of one-time universal screening for chronic hepatitis B infection in adults in the United States. *Clin Infect Dis.* 2022 Jan 29;74:210-217.

Han Y, et al. The incidence of hepatocellular carcinoma in patients with hepatitis B cirrhosis during long-term antiviral therapy: a real world cohort study. *Hepatol Int.* 2021;15(Suppl 1):S45.

Kim H-L, et al. Cost-effectiveness of antiviral treatment in adult patients with immune-tolerant phase chronic hepatitis B. *Gut*. 2021;70:2172-2182.

Lim YS, et al. Impact of expanding antiviral treatment criteria at a population level in the Republic of Korea: a modeling analysis. *Hepatol Int*. 2021;15:S31.

Musabaev E, Sadirova S, Bakieva S, et al. Simplified test and treat protocols for population level screening and elimination of hepatitis B and hepatitis C in Uzbekistan. *J Hepatol*. 2021;75:S642-S643.

Razavi H. Cost-effectiveness analysis of HCV/HBV elimination in the world. Presented at Global Hepatitis Summit, 18 June 2021. Virtual meeting.

Razavi-Shearer D, et al. Cost-effectiveness analysis of treating all HBsAg+ individuals in the United States. *Hepatology*. 2021;74:22A.

Kim D, et al. Guidelines change for antiviral therapy reduced the risk of HBV-related HCC development among cirrhotic patients in South Korea. *J Hepatol*. 2020;73:S880.

Lepers C, et al. Cost-effectiveness of scaling-up treatment with nucleoside analogue (NA) for chronic HBV infection: towards a simplification of recommendations? (ANRS study). *J Hepatol*. 2020;73:S797-S798.

Razavi H, et al. The case for testing and treating all HBV patients. *J Hepatol*. 2020;73:S807.

Sanai FM, et al. A tool to measure the economic impact of hepatitis B elimination: a case study in Saudi Arabia. *J Infect Public Health*. 2020;13:1715-1723.

Oyagüez I, et al. Cost-effectiveness and clinical impact of antiviral strategies of HBeAg-positive and -negative chronic hepatitis B. *Ann Hepatol*. 2017;16:358-365.

Crossan C, et al. Cost-effectiveness of noninvasive liver fibrosis tests for treatment decisions in patients with chronic hepatitis B in the UK: systematic review and economic evaluation. *J Viral Hepat.* 2016;23:139-149.

Post SE, et al. A simulation shows that early treatment of chronic hepatitis B infection can cut deaths and be cost-effective. *Health Aff (Millwood).* 2011;30:340-348.

Liu M, et al. Transition rates to cirrhosis and liver cancer by age, gender, disease and treatment status in Asian chronic hepatitis B patients. *Hepatol Int*. 2021;15:71-81.

Chahal HS, et al. Cost-effectiveness of hepatitis B virus infection screening and treatment or vaccination in 6 high-risk populations in the United States. *Open Forum Infect Dis*. 2018;6:ofy353.

Nayagam S, et al. Requirements for global elimination of hepatitis B: a modelling study. *Lancet Infect Dis*. 2016;16:1399-1408.

Zhang H, et al. Application of Bayesian approach to cost-effectiveness analysis of antiviral treatments in chronic hepatitis B. *PLoS One*. 2016;11:e0161936.

Hulstaert F, et al. Should chronic hepatitis B be treated as early as possible? *Int J Technol Assess Health Care*. 2013;29:35-41.

Robotin M, et al. Cost of treating chronic hepatitis B: comparison of current treatment guidelines. *World J Gastroenterol*. 2012;18:6106-6113.

Enriquez AD, et al. Cost-effectiveness of suppressing hepatitis B virus DNA in immune tolerant patients to prevent hepatocellular carcinoma and cirrhosis. *Aliment Pharmacol Ther.* 2007;26:383-391.
